# Enhanced Wavelet-Convolution and Few-Shot Prototype-Driven Framework for Incremental Identification of Holstein Cattle

**DOI:** 10.3390/s25164910

**Published:** 2025-08-08

**Authors:** Weijun Duan, Fang Wang, Honghui Li, Buyu Wang, Yuan Wang, Xueliang Fu

**Affiliations:** 1 College of Computer and Information Engineering, Inner Mongolia Agricultural University, Hohhot 010018, China; 2020302100004@emails.imau.edu.cn (W.D.); wdwjf68@163.com (F.W.); lihh@imau.edu.cn (H.L.); bywang08@imau.edu.cn (B.W.); 2 National Center of Technology Innovation for Dairy-Breeding and Production Research Subcenter, Hohhot 010018, China; wangyuan@imau.edu.cn; 3 Key Laboratory of Smart Animal Husbandry at Universities of Inner Mongolia Autonomous Region, Hohhot 010018, China; 4 College of Animal Science, Inner Mongolia Agricultural University, Hohhot 010018, China

**Keywords:** Holstein cattle, wavelet convolution, ResWTA, prototype network, incremental identification

## Abstract

Individual identification of Holstein cattle is crucial for the intelligent management of farms. The existing closed-set identification models are inadequate for breeding scenarios where new individuals continually join, and they are highly sensitive to obstructions and alterations in the cattle’s appearance, such as back defacement. The current open-set identification methods exhibit low discriminatory stability for new individuals. These limitations significantly hinder the application and promotion of the model. To address these challenges, this paper proposes a prototype network-based incremental identification framework for Holstein cattle to achieve stable identification of new individuals under small sample conditions. Firstly, we design a feature extraction network, ResWTA, which integrates wavelet convolution with a spatial attention mechanism. This design enhances the model’s response to low-level features by adjusting the convolutional receptive field, thereby improving its feature extraction capabilities. Secondly, we construct a few-shot augmented prototype network to bolster the framework’s robustness for incremental identification. Lastly, we systematically evaluate the effects of various loss functions, prototype computation methods, and distance metrics on identification performance. The experimental results indicate that utilizing ResWTA as the feature extraction network achieves a top-1 accuracy of 97.43% and a top-5 accuracy of 99.54%. Furthermore, introducing the few-shot augmented prototype network enhances the top-1 accuracy by 4.77%. When combined with the Triplet loss function and the Manhattan distance metric, the identification accuracy of the framework can reach up to 94.33%. Notably, this combination reduces the incremental learning forgetfulness by 4.89% compared to the baseline model, while improving the average incremental accuracy by 2.4%. The proposed method not only facilitates incremental identification of Holstein cattle but also significantly bolsters the robustness of the identification process, thereby providing effective technical support for intelligent farm management.

## 1. Introduction

In smart animal husbandry, accurate and convenient individual identification is a prerequisite for achieving large-scale and intelligent management of farms. As the core breed of the global dairy industry, the precise identification of Holstein cattle holds significant importance for precision feeding, health monitoring, genetic breeding, and production management. Currently, the individual identification of Holstein cattle primarily relies on ear tags, which pose significant invasiveness and the risk of detachment. Once tags fall off, it can lead to confusion regarding individual identity, interruptions in production traceability, and failures in genetic information. In recent years, machine vision-based individual identification technology has offered new solutions for the meticulous management of farms due to its non-invasive nature, sustainable monitoring capabilities, and ease of implementation [1].

The unique distribution of hair color patches in Holstein cattle serves as a natural marker for visual individual identification [2,3,4]. However, existing identification methods face several bottlenecks: First, most studies rely on facial and lateral body features for individual identification, which is challenging to apply in practical production environments due to difficulties in feature extraction caused by lens occlusion and motion blur [5,6,7]. Second, many studies operate within a closed-set identification framework, limiting their ability to identify only those individuals who appear in the training set, which hampers adaptability to open scenarios where new individuals continuously enter the scene [8,9]. Additionally, while current mainstream convolutional neural networks can capture local texture details, they exhibit insufficient capability to express the overall structure of the hair patches. Consequently, model performance significantly decreases under abnormal conditions, such as local occlusion, cattle back stains, or random production markers [10,11]. Furthermore, existing open-set identification methods lack the stability required to effectively discriminate new individuals in real production environments and have limited capabilities for online incremental identification [12,13,14,15]. These limitations hinder the application of vision-based individual identification techniques for Holstein cattle in practical farming scenarios.

To address the above challenges, this study proposes an incremental identification framework for Holstein cattle based on a top-view perspective. First, we design a novel feature extraction network, ResWTA, which integrates wavelet convolution and a spatial attention mechanism to enhance the feature expression capability of the overall patch structure of the back by expanding the convolutional sensory field. Second, we construct a few-shot augmented prototype network along with a distance metric module to facilitate the online incremental identification of new individuals. Finally, we systematically evaluate the impacts of various loss functions, prototype computation strategies, and distance metric methods on incremental identification performance to verify the robustness and practicality of the proposed framework in complex scenarios. Experimental results indicate that the proposed method significantly enhances the performance of incremental identification of Holstein cattle individuals from a top-view perspective, providing an efficient and reliable solution for open individual management in pastureland.

The primary contributions of this study are as follows:

(1) We propose a novel feature extraction network, ResWTA, which fuses wavelet convolution with a spatial attention mechanism. This design significantly enhances the model’s ability to extract features from the cattle’s back pattern structure.
(2) We construct a few-shot augmented prototype network aimed at improving the incremental identification performance of the framework in production scenarios.
(3) We introduce an incremental identification framework that integrates the feature extraction network, the prototype network, and a distance metric module. Through evaluating the impact of each component on the incremental identification performance of Holstein cattle, we identify the optimal incremental identification framework, thereby providing an efficient and practical individual identification solution for the smart animal husbandry industry.

## 2. Related Work

Currently, deep learning-based individual identification methods for Holstein cattle primarily rely on natural biometric features such as the face [5,16,17], muzzle [6,7,18,19], flank [20,21,22,23], and rump [24] for characterization and matching. However, identification based on the cattle’s face requires high image quality and is adversely affected by the cattle’s frequent head movements, complicating data acquisition. Identification based on the texture of the mouth and nose requires clear images taken at close range, which may induce stress reactions in the cattle. While flank and rump images contain rich individual features, they must be captured from a horizontal perspective, making them susceptible to obstructions, motion blur, and skin covering, resulting in unstable features. In contrast, dorsal images of Holstein cattle are particularly suitable for individual identification in production environments due to their distinctive characteristics, stable exposure, and minimal occlusion, which can be easily obtained from a top-view perspective.

In recent years, scholars have conducted research on the individual identification of Holstein cattle from a top-view perspective. Zhao et al. (2019) proposed a visual identification system for Holstein cattle utilizing top-view images, wherein features are extracted and matched from the dorsal image using scale-invariant feature transform (SIFT), fast feature point detection (FAST), and binary robust invariant scalable keypoints (BRIEF), achieving an identification accuracy of 96.72% [25]. However, it relied on manually designed feature point selection algorithms, which may result in inaccurate feature point detection when images are blurred or when individual occlusions occur, subsequently affecting identification accuracy. Bello et al. (2020) introduced an individual identification scheme based on the back pattern of cattle through convolutional neural networks (CNNs) and deep belief networks (DBNs) for feature extraction and classification, achieving an identification accuracy of 89.95% on a multi-breed dataset [26]. Ferreira et al. (2022) evaluated the capability of a deep learning model to identify individual Holstein calves across various postures and growth cycles using 3D images of cattle backs, attaining F1-scores as high as 95.9% in closed-set evaluations [27]. Ma et al. (2025) developed a lightweight feature extraction network, CowBackNet, by integrating deep separable convolution, inverse residual structures, and multiple attention mechanisms, achieving an identification accuracy of 95.86% on the Cows2021 dataset [28]. Although the aforementioned methods have demonstrated strong performance under closed-set conditions, they struggle in open breeding scenarios where new individuals are continuously introduced. Furthermore, most of these approaches are based on convolutional neural networks, which primarily focus on local detail information and often fail to adequately represent the overall structure of the mottled pattern. Consequently, their performance significantly declines under abnormal conditions such as local occlusions, dirt on the cattle’s back, and production markers.

In the field of open-set identification, several scholars have proposed open-set identification frameworks for Holstein cattle based on the principles of metric learning. Andrew et al. (2021) utilized ResNet to extract dorsal mottled features of cattle and constructed a discriminative embedding space, achieving an open-set identification accuracy of 93.75% [12]. Gao et al. (2022) integrated self-supervised learning, metric learning, and active learning, resulting in an enhancement of open-set identification accuracy from 74.9% to 92.44% on the Cows2021 dataset [13]. Wang et al. (2024) attained an identification accuracy of 94.26% by merging a lightweight feature extraction network with a distance metric [14]. Additionally, Wang et al. (2024) improved identification accuracy for the Cows2021 dataset by introducing a spatial feature transformation module to address the impact of variations in cattle posture and orientation on individual identification in a production environment, achieving an accuracy of 94.31% [15]. Despite these advancements, the aforementioned studies highlight persistent issues, such as instability in category discrimination and limited online incremental identification capabilities, which hinder the ability to maintain stable identification of new individuals continuously introduced in actual production environments.

In recent years, image enhancement techniques have been widely applied in deep vision tasks. Methods such as denoising, rotation, cropping, blurring, brightness adjustment, and noise injection have been employed to improve image quality or augment the sample space, thereby effectively enhancing model robustness and generalization [29,30,31]. However, existing studies have mainly focused on the role of image enhancement in improving model generalization during the training phase, while the feasibility of applying image enhancement to enhance prototype discriminability during the prototype construction stage of incremental identification has not yet been investigated.

In summary, existing studies exhibit significant deficiencies in feature expression, adaptability to open scenes, and stability in incremental identification. To address these issues, this study proposes an incremental identification framework utilizing the feature extraction network ResWTA and a few-shot augmented prototype network. This framework enhances the low-frequency feature expression of the overall dorsal patch structure of cattle while simultaneously achieving robust online incremental identification of new individuals. Thus, it effectively fills the research gap concerning the incremental identification of Holstein cattle in the open scene of a top-view perspective.

## 3. Materials and Methods

### 3.1. Dataset

#### 3.1.1. Data Acquisition

**Figure 1:**
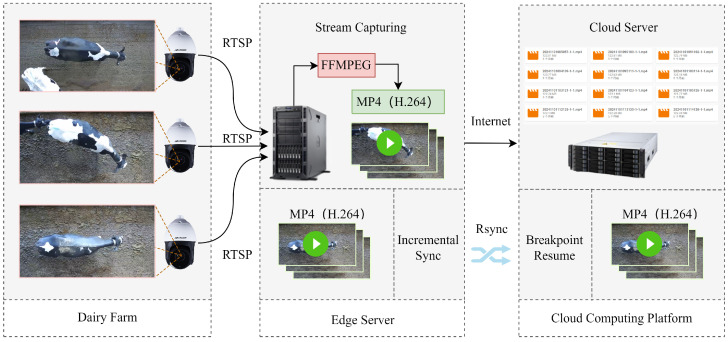
Data acquisition scheme. The camera was installed at the top of the barn, providing a top-view perspective. The video streams were captured using FFMPEG and stored as video files on an edge server. These files were subsequently synchronized incrementally to the cloud platform using RSYNC.

This study selected a large-scale dairy farm near Hohhot City, Inner Mongolia Autonomous Region, as the data collection site. Video recordings of adult Holstein cattle were conducted from 7–20 December 2023, under natural daylight conditions. The acquisition equipment consisted of a DS-2DC4223IW-D dome camera (Hikvision, Hangzhou, China) featuring a horizontal rotation of 360°, a vertical rotation from −15° to 90°, a 23× optical zoom, a 16× digital zoom, with a focal length of 8 mm, and a field of view of 57.6°. The camera was mounted 4.3 m above the ground at the top of the barn, providing a vertical overhead view of the Holstein cattle passage area for video capture, with a resolution of 1920 × 1080 pixels and a frame rate of 25 frames per second. RTSP video streams were recorded on-site utilizing FFMPEG (V4.1) software on a PowerEdge T420 edge server (Dell, Round Rock, TX, USA). An MP4 (H.264) file was generated every 10 min, and the local data were subsequently synchronized to the cloud computing platform via RSYNC (V3.1.3). Figure 1 illustrates the data acquisition scheme.

#### 3.1.2. Data Preprocessing

To mitigate the impact of redundant data on model training resulting from the significant number of duplicate samples generated by frame-by-frame image extraction and the brief pauses or slow movements of Holstein cattle, the following pre-processing was implemented on the original video data in this study:

(1) Video frame extraction: The video files were processed using FFMPEG (V4.1) software to generate image data by extracting one frame for every five frames. This method effectively reduced data redundancy while maintaining an adequate training sample size.
(2) Image de-weighting based on the SSIM: The Structural Similarity Index (SSIM) algorithm was employed to assess the similarity of adjacent images, with a threshold set at 0.79 to eliminate highly similar data.
(3) Image cropping: GIMP (V2.10.36) was utilized to crop the filtered images, retaining only the back region of a single Holstein cattle.
(4) Image annotation: The cropped images were manually labeled and classified to provide reliable data labels for subsequent model training and performance evaluation.

After completing these pre-processing operations, a self-acquisition dataset comprising back images of 49 Holstein cattle was constructed in this study.

#### 3.1.3. Dataset Construction

To further enhance the diversity of the data, this study introduced the public datasets Cows2021 [32] and MultiCamCows2024 [8] for data expansion. Initially, samples with solid color backs were excluded. Subsequently, all images were rotated to ensure horizontal alignment, with the cattle’s heads consistently facing right. This adjustment aimed to eliminate the interference of orientation differences during feature extraction, thereby improving the discriminative power and consistency of the dataset. Ultimately, 176 Holstein cattle samples were selected from the Cows2021 dataset, along with 77 Holstein cattle samples captured by multiple cameras from the MultiCamCows2024 dataset.

The experimental data size is presented in Table 1. Initially, 3940 images of 10% (30) Holstein cattle were randomly selected from the total sample to create a test set for evaluating the model’s incremental identification performance. Subsequently, the remaining 272 Holstein cattle’s images were randomly divided into a training set and a validation set in an 8:2 ratio, with the training set containing 33,775 images and the validation set including 8443 images.

**Figure 2:**
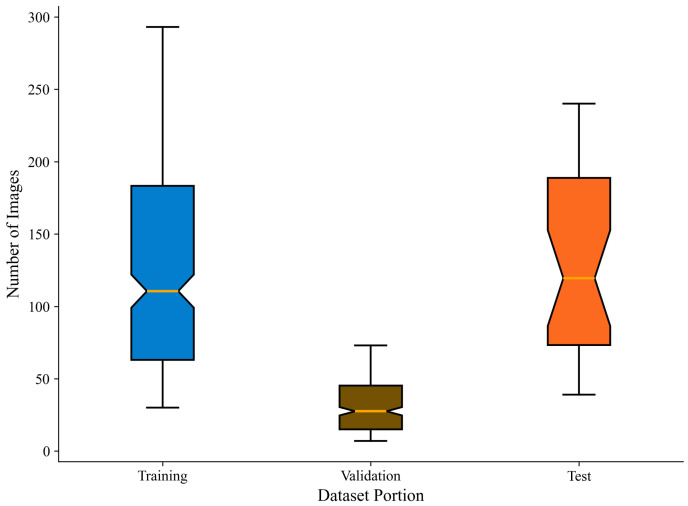
Distribution map of the number of images in the dataset. The box-and-line plot illustrates the distribution of Holstein cattle instances, with the median (orange line) and interquartile range highlighting the concentration trend and mid-range variation in the sample. Additionally, the box-and-whisker plot reveals the long-tailed nature of the distribution.

Figure 2 illustrates the distribution of sample sizes within the datasets. The figure illustrates that a majority of Holstein cattle possess adequate training samples, enabling the model to learn diverse patch features. Additionally, the dataset maintains a long-tail distribution of both few-sample and multi-sample instances, which enhances the model’s generalization capability across varying sample sizes. The box plot and median of the test set are highly consistent with those of the training set, indicating that the test set can effectively reflect the diversity of the overall dataset in terms of sample distribution.

In open-set identification methods, it is typically necessary to calculate the metrics of input images to ascertain whether two images belong to the same subject. The few-shot augmented prototype network designed in this study first computes the prototype representation based on five images of the same subject and then compares it with another validation image to determine if they depict the same subject. Specifically, for each Holstein cattle in the test set, five images were randomly selected as prototype samples. Subsequently, one image from the remaining images of the same cattle was randomly chosen as a validation sample, while one image from each of the remaining 29 cattle was randomly selected as a negative sample. To ensure the robustness of the experimental results, this process was repeated ten times, resulting in a total of 300 sets of positive samples and 300 sets of negative samples constructed for each of the 30 cattle.

### 3.2. Framework Overview

**Figure 3:**
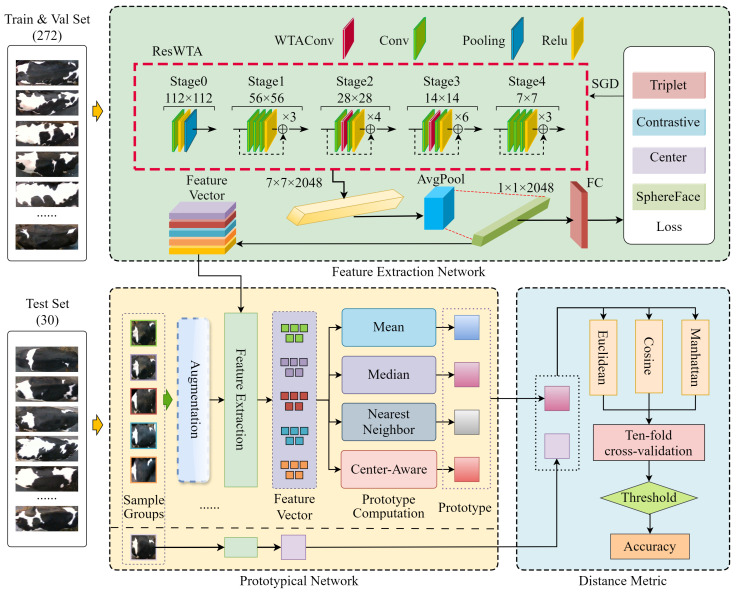
Structure diagram of individual incremental identification framework for Holstein cattle.

This paper addresses the challenges posed by closed-set identification in breeding scenarios where new individuals continuously join, as well as the inadequacies of existing open-set identification methods in complex production environments. To tackle these issues, we propose an incremental identification framework, which comprises three components: the feature extraction network ResWTA, the few-shot augmented prototype network, and the distance metric module. Initially, the feature extraction network extracts discriminative feature vectors from cattle back images. Subsequently, the prototype network employs a few-shot augmented strategy to dynamically generate prototype representations for each individual during the incremental stage. Finally, the distance metric module computes the similarity between the prototypes and the samples to be recognized, utilizing a selected metric function to facilitate online incremental identification. The overall structure is illustrated in Figure 3.

In the model training stage (feature extraction network section of Figure 3), this study constructs a feature extraction network, ResWTA, based on ResNet50. Specifically, the feature extraction module, WTAConv, which integrates wavelet convolution and a spatial attention mechanism, replaces the 3×3 convolutions in Stage 2 and Stage 3 of ResNet50 to enhance the representation of the overall texture structure of the cattle’s back. End-to-end training is performed on the training set, mapping the input image into a 1×1×2048 depth feature vector.

In the prototype construction stage (prototypical network part of Figure 3), a set of 5 images from the positive and negative sample groups is randomly selected as prototype samples. These images undergo data enhancement in 4 different modes, generating a total of 20 samples. The 25 samples are then input into the ResWTA network to extract 25 feature vectors of size 1×1×2048. Finally, the 25 feature vectors are aggregated using a predefined prototypical computation method to construct the prototype representation of the category.

In the incremental identification stage (distance metric section of Figure 3), the prototype representations obtained through calculations are compared with the feature vectors extracted from the validation samples via ResWTA for distance measurement. By comparing with a preset threshold, it is determined whether the validation samples and prototype samples belong to the same category, achieving online incremental identification.

### 3.3. Feature Extraction Network

**Figure 4:**
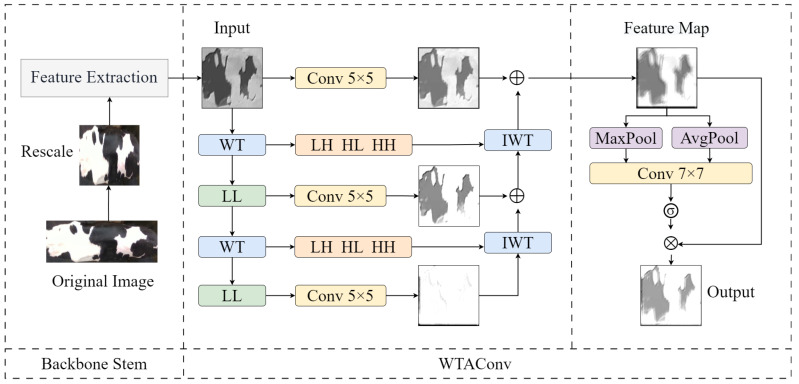
Structure diagram of WTAConv.

The classification task utilizing cattle back images relies significantly on low-frequency features, including back patterns. Existing research indicates a strong correlation between the classifier’s open-set decision capability and its accuracy in closed-set decisions [33]. Thus, effectively extracting low-frequency features, such as the patterns on the cattle’s back, is crucial for designing an open-set framework. In this study, we propose a novel feature extraction network, ResWTA, based on ResNet50. This network incorporates WTAConv, a convolutional module that integrates wavelet convolution with a spatial attention mechanism, facilitating the adaptive enhancement of features in the cattle back pattern region. The structure of WTAConv is illustrated in Figure 4.

Initially, the feature map $X\in R^{C\times H\times W}$ is input into the WTAconv, where a three-level wavelet decomposition is performed, as illustrated in Equation (1). This process generates three low-frequency subbands and nine high-frequency subbands at varying scales, allowing for the extraction of both global and detailed information across different scales.

(1) $$\left[X_{LL}^{\left(i\right)},X_{LH}^{\left(i\right)},X_{HL}^{\left(i\right)},X_{HH}^{\left(i\right)}\right]=WT\left(X_{LL}^{\left(i-1\right)}\right)\;$$

where i=1,2,3, $X_{LL}^{\left(0\right)}=X$, WT(·) represents the single level two-dimensional discrete wavelet transform operation; $X_{LL}^{\left(i\right)}$, $X_{LH}^{\left(i\right)}$, $X_{HL}^{\left(i\right)}$ and $X_{HH}^{\left(i\right)}$ represent the low-frequency subband, the horizontal high-frequency subband, the vertical high-frequency subband, and the diagonal high-frequency subband features extracted after the *i*-th layer decomposition, respectively.

Secondly, to enhance the structural information of the cattleback pattern, convolution is performed only on the low-frequency subband $X_{LL}^{\left(i\right)}$ at each layer, while the high-frequency subbands ($X_{LH}^{\left(i\right)}$, $X_{HL}^{\left(i\right)}$, $X_{HH}^{\left(i\right)}$) are retained without modification. At each layer, the current low-frequency convolutional feature $Y_{LL}^{\left(i\right)}$ is summed and fused with the reconstructed output $Z^{\left(i+1\right)}$ from the previous layer. This combined output, along with the high-frequency features $X_{LH}^{\left(i\right)}$, $X_{HL}^{\left(i\right)}$, $X_{HH}^{\left(i\right)}$, is then input into the inverse wavelet transform module IWT(·) to yield the reconstructed feature $Z^{\left(i\right)}$ at the current layer, as detailed in Equation (2). This process is iterative and progresses from the bottom layer to the top. At the highest layer (i=1), a fused feature $Z^{\left(1\right)}$ is obtained, which incorporates multi-layer low-frequency convolutional features along with high-frequency details. To further integrate shallow feature information, a 5×5 convolution operation is introduced in parallel with the main branch to extract local detail information from X. This information is subsequently fused with the final reconstructed feature $Z^{\left(1\right)}$ to produce the spatial feature map.

(2) $$Z^{\left(i\right)}=IWT\left(Y_{LL}^{\left(i\right)}+Z^{\left(i+1\right)},X_{LH}^{\left(i\right)},X_{HL}^{\left(i\right)},X_{HH}^{\left(i\right)}\right),\;Z^{\left(4\right)}=0$$

where IWT(·) denotes the two-dimensional inverse wavelet transform operation; $Y_{LL}^{\left(i\right)}$ represents the convolution features of the low-frequency subband at the *i*-th layer; $Z^{\left(i+1\right)}$ is the reconstructed output of the previous layer; and $Z^{\left(i\right)}$ is the reconstructed result of the current layer, which is initially set to $Z^{\left(4\right)}=0$.

Finally, the spatial attention module performs both maximum pooling and average pooling on the spatial feature map. It concatenates the results of these two pooling operations along the channel dimension and feeds them into a 7×7 convolutional layer to generate the spatial attention map. This map is then applied to weight the original feature map using a sigmoid function, thereby enhancing the salient regions, such as the cattle’s back pattern, while suppressing the background regions. The input feature map is denoted as $Z^{\left(i\right)}\in R^{C\times H\times W}$, and the output feature map is represented as ${Z^{\left(i\right)}}^{\prime }$. Equation (3) illustrates the calculation process.

(3) $${Z^{\left(i\right)}}^{\prime }=\sigma \left(f^{7\times 7}\left(\left[\mathrm{AvgPool}\left(Z^{\left(i\right)}\right);\;\mathrm{MaxPool}\left(Z^{\left(i\right)}\right)\right]\right)\right)⊙Z^{\left(i\right)}$$

where $\mathrm{AvgPool}(Z^{\left(i\right)})$ and $\mathrm{MaxPool}(Z^{\left(i\right)})$ represent the feature maps after average pooling and max pooling, respectively; $\left[\mathrm{AvgPool}\left(Z^{\left(i\right)}\right);\;\mathrm{MaxPool}\left(Z^{\left(i\right)}\right)\right]$ indicates the concatenation operation; $f^{7\times 7}\left(\cdot \right)$ represents the convolution operation with a kernel size of 7×7; σ(·) represents the sigmoid activation function.

It has been established that the intermediate stages of deep networks play a crucial role in facilitating the transition from low-level to high-level features, significantly influencing the overall performance of the network [34]. This study selects ResNet50, which demonstrated the best performance among various classification networks, as the foundational architecture, and then employs WTAConv to replace the 3×3 convolutional operations in Stage 2 and Stage 3 of ResNet50, thereby enhancing the network’s capacity to extract low-frequency and global features. This modification develops the ResWTA backbone network that is appropriate for extracting Holstein cattle back features. In Stage 2, we utilize a three-level wavelet decomposition, while in Stage 3, a two-level wavelet decomposition is applied. Additionally, we implement a convolution with a kernel size of 5×5 to regulate computational load and mitigate excessive loss of spatial information.

### 3.4. Loss Function

In this study, four loss functions—Triplet loss [35], Contrastive loss [36], Center loss [37], and SphereFace loss [38]—are selected to optimize the discriminative properties of features, considering the specific requirements of the individual identification task for Holstein cattles regarding category increment and distance metrics.

The Triplet loss function enhances intra-class and inter-class distances by constructing a Triplet consisting of anchor samples, positive samples, and negative samples, as illustrated in Equation (4).

(4) $$L_{\mathrm{Triplet}}\left(a,p,n\right)=\max\left({0,∥f\left(a\right)-f\left(p\right)∥}_{2}^{2}-{∥f\left(a\right)-f\left(n\right)∥}_{2}^{2}+\alpha \right)$$

where *a* is an anchor sample; *p* is the positive sample of the same category as *a*; and *n* is the negative sample of a different category from *a*. The function f(·) maps samples to the embedding space, and α>0 is a preset interval hyperparameter.

The Contrastive loss function explicitly constrains the minimization of distance for similar samples and the maximization of distance for dissimilar samples in the embedding space by modeling the similarity between pairs of samples, as illustrated in Equation (5).

(5) $$L_{\mathrm{contrastive}}=\frac{1}{2N}\sum_{i=1}^{N}\left[y_{i}\;d_{i}^{2}+\left(1-y_{i}\right)\;{\left(\max\{\gamma -d_{i},\;0\}\right)}^{2}\right]\;$$

where *N* denotes the total number of sample pairs; $y_{i}\in \left\{0,1\right\}$ is the labeling indication of the *i*-th pair of samples, with a value of 1 for similar and 0 for dissimilar; $d_{i}$ is the Euclidean distance of the *i*-th pair of samples in the embedding space; and γ>0 is the safety interval (margin) parameter for the contrast loss.

The Center loss function regulates the class center by consistently minimizing the distance between intra-class samples and the class center. Refer to Equation (6).

(6) $$L_{\mathrm{center}}=\frac{1}{2N}\sum_{i=1}^{N}{∥f\left(x_{i}\right)-c_{y_{i}}∥}_{2}^{2}$$

where $c_{y_{i}}$ denotes the category center of the category $y_{i}$ to which the sample $x_{i}$ belongs.

The SphereFace loss function enhances the distinction between classes in the embedding space by increasing the angular distance between feature vectors, as illustrated in Equation (7).

(7) $$L_{\mathrm{sphere}}=-\frac{1}{N}\sum_{i=1}^{N}\log\frac{e^{\;s\;\cos\left(m\;\theta_{y_{i},i}\right)}}{e^{\;s\;\cos\left(m\;\theta_{y_{i},i}\right)}+\sum_{j\neq y_{i}}e^{\;s\;\cos\left(\theta_{j,i}\right)}}$$

where *s* is the scale factor, *m* is the angular interval, and $\theta_{j,i}$ denotes the angle between the *i*-th sample and the center of the *j*-th category.

### 3.5. The Prototypical Network

In the breeding scene, the introduction of new individuals often leads to the acquisition of only a limited number of single-scene samples, which can result in inadequate feature representation. This study proposes a prototype network aimed at enhancing few-shot datasets through image enhancement, feature extraction, and prototype computation.

**Figure 5:**
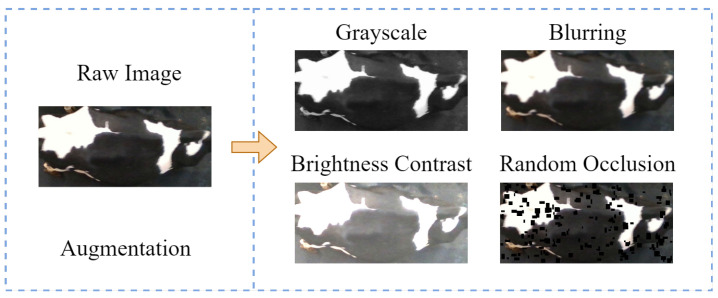
Example of image enhancement.

First, four data enhancement strategies are applied to five prototype samples from each group of positive and negative samples to simulate imaging interference in the production environment. These strategies include grayscaling, blurring (with kernel sizes randomly selected from [3, 7] and standard deviations ranging from [0.1, 2.0]), brightness and contrast adjustment (with brightness coefficients adjusted linearly within [−0.2, 0.2] and contrast scaled randomly between [0.8, 1.2]), and random occlusion of small targets (by generating squares with widths and heights between [10, 16] pixels, assigned a grayscale value of 0), as illustrated in Figure 5. Subsequently, five prototype samples along with the corresponding twenty enhanced samples are input into the feature extraction network ResWTA to obtain twenty-five feature vectors of dimensions 1×1×2048. Finally, the 25 feature vectors are aggregated through four methods—mean, median, nearest neighbor, and center-aware—to determine their category prototypes.

In this study, three distance measures—Manhattan, Cosine, and Euclidean—are selected to assess the similarity between the prototype representation and the validation samples. To mitigate the potential bias introduced by a single data division, the experiment employs the ten-fold cross-validation method. This approach determines the optimal distance thresholds in the training set of each fold and evaluates model performance in the validation set, thereby ensuring the scientific rigor and robustness of the threshold selection.

The 600 sets of positive and negative samples in the test set are randomly divided into 10 subsets. Nine subsets are sequentially selected for threshold determination, while one subset is reserved for calculating the top-1 accuracy under the established threshold. Initially, the threshold ranges for Manhattan, Cosine, and Euclidean distances are set to $d_{\mathrm{man}}\in \left[0,100\right]$, $d_{\cos}\in \left[0,1\right]$, and $d_{\mathrm{euc}}\in \left[0,15\right]$, respectively. Exhaustive searches are conducted in increments of 0.1, 0.0001, and 0.01. Subsequently, for each fold subset, the top-1 accuracy is computed for each candidate threshold value, and the threshold $d_{i}^{\mathrm{best}}$ that maximizes the top-1 accuracy for that fold is recorded as the threshold for this round of computation. Finally, after completing 10 rounds of validation, the 10 thresholds and their corresponding highest top-1 accuracies are obtained, and the globally optimal threshold τ is computed using the weighted average method, as seen in Equation (8).

(8) $$\tau =\frac{\sum_{i=1}^{10}{\mathrm{Acc}}_{i}\cdot d_{i}^{\mathrm{best}}}{\sum_{i=1}^{10}{\mathrm{Acc}}_{i}}$$

where ${\mathrm{Acc}}_{i}$ is the highest top-1 accuracy of the *i* fold validation, and $d_{i}^{\mathrm{best}}$ is the corresponding optimal threshold.

### 3.6. Evaluation Indicators

To effectively evaluate the performance of the proposed framework for the incremental identification of Holstein cattle individuals, this study employed top-1 and top-5 accuracy to assess the model’s classification performance, along with average incremental accuracy (AIA) and forgetfulness (F) to evaluate the effect of incremental identification.

The top-1 accuracy is calculated by determining whether the category with the highest confidence in the prediction result matches the true label. This metric evaluates the classification performance of the model based on its single best prediction, as illustrated in Equation (9). In contrast, top-5 accuracy gauges the model’s capability to include true labels within the top five predicted categories.

(9) $$\mathrm{Top}-1\;\mathrm{Accuracy}=\frac{TP}{TP+FN}$$

where TP refers to the count of samples accurately identified by the model as belonging to the positive category, while FN denotes the count of samples erroneously classified by the model as belonging to the negative category.

The average incremental accuracy (AIA) serves as a metric for evaluating the overall performance of a model in identifying all known individuals, as unknown individuals are continuously introduced. Assuming the model progresses through *T* incremental stages, with the classification accuracy at stage *i* represented as ${\mathrm{Acc}}_{i}$, the AIA is computed as shown in Equation (10).

(10) $$AIA=\frac{1}{T}\sum_{i=1}^{T}Acc_{i}$$

The forgetfulness (F) quantifies the extent to which the model forgets previously learned knowledge during subsequent training stages. The calculation is articulated in Equation (11).

(11) $$F=\frac{1}{T-1}\sum_{t=2}^{T}\left(\max_{l<t}A_{l}^{l}\;-\;A_{l}^{t}\right)$$

where $A_{l}^{t}$ represents the detection accuracy of the categories from the previous stage after the *t*th stage of training.

## 4. Results

### 4.1. Experimental Environment and Parameter Settings

The experiments were conducted on a server running Ubuntu Server 22.04, equipped with dual Intel(R) Xeon(R) Gold 6139M CPUs operating at 2.30 GHz, 128 GB of RAM, and eight NVIDIA GeForce RTX 3090 graphics cards (NVIDIA, Santa Clara, CA, USA). The software environment incorporates deep learning frameworks, including Python 3.10.11, CUDA 11.7, and PyTorch 2.0.1. During the model training phase, the stochastic gradient descent optimization strategy was employed, featuring an initial learning rate of 0.02, a momentum parameter of 0.9, a weight decay coefficient of 0.0001, and a batch size of 64. For a comprehensive overview of the experimental parameter settings, please refer to Table 2.

### 4.2. Comparison of Feature Extraction Network Performance

**Figure 6:**
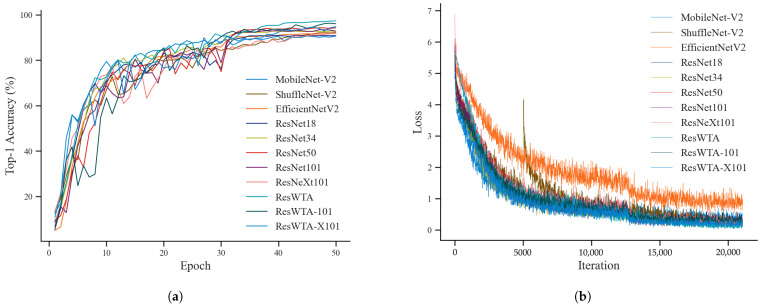
Top-1 accuracy and loss change curves during training. (**a**) Top-1 accuracy; (**b**) Loss.

To verify the effectiveness of the WTAConv convolution and identify the optimal feature extraction network, this study conducted a comparative experiment involving various feature extraction networks. Eleven models were selected—MobileNet-V2, ShuffleNet-V2, EfficientNet-V2, ResNet18, ResNet34, ResNet50, ResNet101, ResNeXt101—and the models proposed in this paper—ResWTA (derived from ResNet50), ResWTA-101 (derived from ResNet101), and ResWTA-X101 (derived from ResNeXt101). All models were initialized with ImageNet-1k pre-training weights obtained from the mmpretrain repository and were subsequently fine-tuned. The models underwent training using the dataset presented in Table 1, with detection accuracy and loss values on the validation set computed after each training round. Figure 6 illustrates the changes in top-1 accuracy and loss values for each model throughout the training process.

As illustrated in Figure 6, the top-1 accuracy of the 11 networks exhibits a general upward trend with varying degrees of fluctuation in the initial training phase. After 30 epochs of training, this accuracy begins to stabilize, and by approximately 35 epochs, ResWTA’s top-1 accuracy exceeds that of the other models, resulting in superior performance. During the model training process, the loss value exhibits a significant decrease and subsequently stabilizes after 5000 iterations.

As shown in Table 3, the top-1 accuracy of lightweight networks such as MobileNet-V2, ShuffleNet-V2, and EfficientNet-V2 is generally low. In contrast, the accuracy of residual networks, including ResNet18, ResNet34, ResNet50, ResNet101, and ResNeXt101, initially increases and then decreases as the number of network layers deepens, with ResNet50 achieving an accuracy of 94.41%. The overall performance of the proposed ResWTA in this study is superior, with a top-1 and top-5 accuracy of 97.43% and 99.54%, respectively, which are 3.02% and 1.67% higher than those of the baseline model, ResNet50, and also surpass the performance of ResWTA-101 and ResWTA-X101.

### 4.3. Experimental Results for Incremental Identification

This paper systematically evaluates the effectiveness of the proposed method in incremental identification tasks by comparing and analyzing the impact of various combinations of four loss functions, four prototype calculation methods, and three distance metric methods on open-set identification performance. The experimental results indicate that the integration of the Triplet loss function, median prototype calculation method, and Manhattan distance metric produces optimal incremental identification performance. Table 4 illustrates that the incremental identification framework utilizing ResWTA attained an accuracy of 94.33% on the test set, reflecting an enhancement of 1.86 percentage points over the conventional ResNet50 incremental identification framework.

### 4.4. Ablation Experiment

Ablation experiments were conducted on the core variables, and the results are presented in Table 5. The implementation of ResWTA results in a 2.33 percentage point increase in accuracy relative to ResNet50. Additionally, the incorporation of the median prototype calculation method yields a 3.63 percentage point improvement, while the application of image enhancement strategies contributes a 1.14 percentage point increase. The optimization of the feature extraction network, prototype mechanism, and image enhancement strategy significantly enhances the accuracy of incremental identification.

### 4.5. Evaluation of the Effectiveness of Incremental Identification

**Figure 7:**
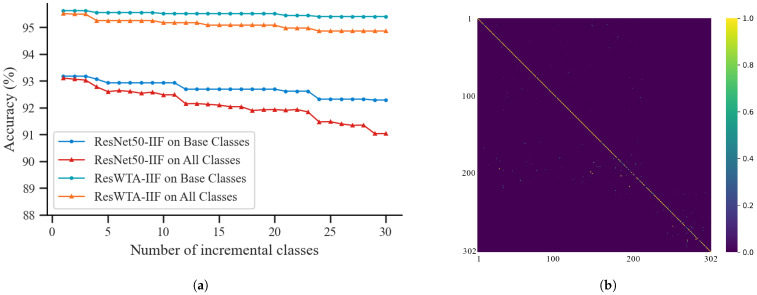
The effect of incremental identification. (**a**) Effect of incremental identification using ResNet50 and ResWTA; (**b**) heat map of the ResWTA confusion matrix.

The experiments evaluate the incremental identification performance of the framework utilizing ResNet50 and ResWTA models independently; the results are presented in Figure 7 and Table 6. The Base Classes depicted in the figure denote the categories on which the feature extraction network has been trained, specifically the validation set. The alteration in identification accuracy concerning Base Classes assesses the framework’s capacity to preserve the identification of original categories following the introduction of new categories, indicating the model’s extent of forgetting. All Classes denote the complete set of categories, encompassing both validation and test sets. The variation in accuracy on this dataset indicates the model’s adaptability and generalization capabilities in identifying categories overall.

Analyzing Figure 7a, it is evident that the identification accuracy of both frameworks exhibits a decline to a certain extent with an increase in the number of incremental categories. This decline is primarily attributed to the degradation of the discriminative properties of existing categories and the misidentification of new categories. In contrast, the ResWTA-based framework demonstrates superior identification accuracy across both evaluation metrics, with a smaller fluctuation in accuracy, indicating more stable performance. Analyzing in conjunction with Table 6, within the Base Classes, the identification accuracy of the framework utilizing ResWTA consistently remains above 95.40%, peaking at 95.62% and dipping to a minimum of 95.40%, with a forgetfulness rate of 2.24%. Conversely, the identification accuracy of the framework employing ResNet50 declines from 93.17% to 92.28%, accompanied by a forgetfulness rate of 7.13%. In the overall identification task encompassing All Classes, the identification accuracy of the ResWTA-based framework decreases from 95.51% to 94.86%, yielding an average incremental accuracy of 95.1%. In contrast, the ResNet50 framework experiences a reduction in maximum identification accuracy from 93.1% to 91.02%, resulting in a significantly lower average incremental accuracy of only 92.7%. Figure 7b presents the confusion matrix heatmap derived from the ResWTA framework. The main diagonal of the image is distinctly highlighted, while the off-diagonal areas demonstrate low confusion characteristics. This suggests that the ResWTA framework possesses robust category differentiation capabilities and stable overall identification performance.

## 5. Discussion

Table 3 indicates that ResWTA demonstrates a 3.02% enhancement in top-1 accuracy relative to the baseline model. ResWTA-101 and ResWTA-X101 demonstrate differing levels of enhancement in top-1 accuracy relative to the networks prior to improvement. This phenomenon is ascribed to the wavelet convolution of the WTAConv module, which delineates low-frequency contours and overarching pattern topologies of the cattle’s back. Additionally, the spatial attention mechanism emphasizes critical pattern detail while mitigating background interference. This aligns with the findings of Finder et al. (2024), which indicate that wavelet convolution can enhance the effective receptive field of the network and improve feature extraction capabilities [39].

Analysis of Table 3 indicates that as the number of model parameters increases, the identification performance of the network gradually improves. However, the identification accuracy of deep networks such as ResNet101 and ResNeXt101 is lower than that of ResNet50, and the classification accuracy of ResWTA-101 and ResWTA-X101 is also lower than that of ResWTA. This phenomenon, often referred to as ’larger model but lower performance’, is closely related to model overfitting. The backs of Holstein cattle targeted in this study possess relatively simple and well-defined texture features. Consequently, the high capacity of deep networks may lead to fitting random noise or chance patterns from a limited number of samples, which ultimately diminishes their generalization performance. This observation aligns with Zhang et al. (2016), who found that high-capacity deep networks are prone to fitting noise in datasets characterized by simple feature structures or low sample complexity, resulting in degraded generalization performance [40]. Furthermore, Janik et al. (2021) demonstrated through effective dimensionality versus complexity entropy metrics that shallow or moderately deep networks can more efficiently capture primary feature patterns, while indiscriminately increasing model depth significantly heightens the risk of overfitting [41]. Similarly, Basha et al. (2019) found that for datasets with low feature dimensions and strong structural regularities, shallow or medium-depth networks outperform deeper models in terms of classification performance [42].

In the field of individual identification based on cattle body patterns, several empirical results align with the findings of this paper. Wang et al. (2023) [43], Hou et al. (2021) [24], and Ma et al. (2025) [28] demonstrated in their studies on individual cattle identification that a lower number of parameters and moderate network depth tend to yield better generalization abilities. Wang et al. (2024) [14] demonstrated that ResNet18 surpassed ResNet50 in the specific task of individual cattle back identification. This further substantiates that in low-complexity visual classification tasks, such as cattle back patterns, augmenting the model’s parameters and depth does not inherently result in performance improvements.

**Figure 8:**
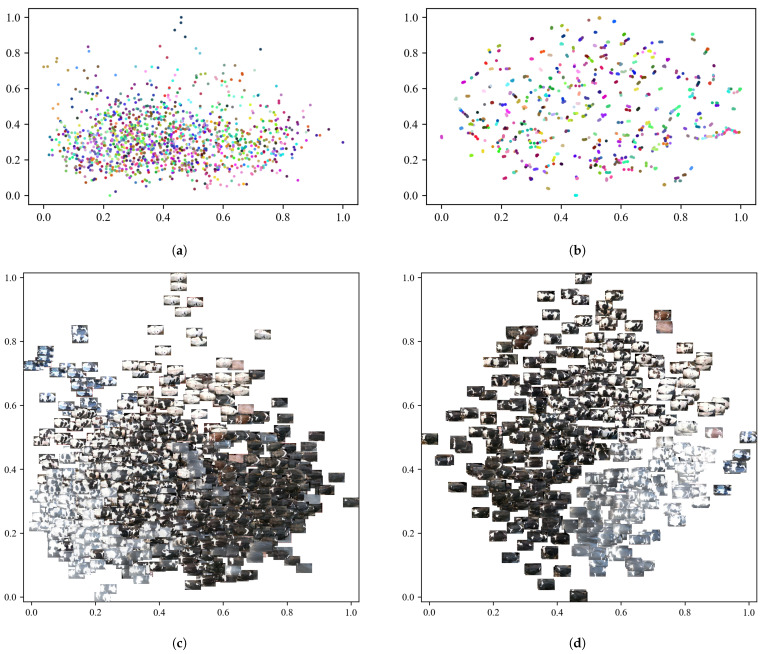
Results of the t-SNE feature dimensionality reduction analysis on the validation set. Different color dots represent different individuals. (**a**) The visualization results before the ResWTA training; (**b**) the visualization results after the ResWTA training; (**c**) the image visualization results before the ResWTA training; (**d**) the image visualization results after the ResWTA training.

This study utilizes the t-SNE dimensionality reduction visualization technique to analyze the feature distribution of the feature extraction network before and after training, as illustrated in Figure 8. The feature points extracted by the untrained ResWTA network are initially distributed in a disordered manner, making it challenging to differentiate between individuals. In contrast, post-training, the sample categories in the feature space demonstrate distinct clustering, with samples of the same class closely converging and those from different classes being effectively separated, exhibiting clear boundaries. This observation aligns with the results of the qualitative experiments presented in Table 3, indicating that the ResWTA network is capable of extracting more discriminative features related to the cattle’s back.

**Figure 9:**
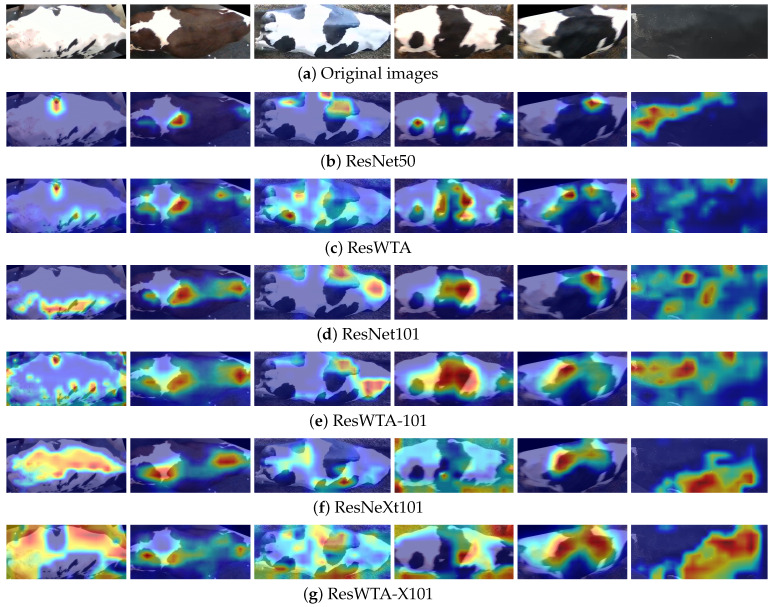
Comparison of class activation maps generated by different models.

This research employs the LayerCAM algorithm to visualize heatmaps corresponding to the outputs of ResNet50, ResNet101, ResNeXt101, and the enhanced model Stage4, as illustrated in Figure 9. Analysis indicates that the model incorporating the WTAConv module exhibits enhanced feature activation in the critical texture regions of the cattle’s back relative to the original model. Furthermore, the activated regions align more closely with the actual pattern boundaries, reflecting a more robust overall focus. This demonstrates that WTAConv can effectively enhance the representation of cattle back patterns.

Further analysis indicates that the identification performance of ResNet101, ResNeXt101, and their variants is inferior to that of ResNet50 and ResWTA as the number of model parameters increases. The primary reason is that the feature structure of cattle back patterns is straightforward and predominantly depends on low-frequency global information for individual identification, and the increased model capacity can easily result in overfitting to non-discriminative noise, as evidenced by the first, third, and fifth images in Figure 9d, and the first, fourth, and sixth images in Figure 9f, which demonstrate overfitting to irrelevant or noisy regions, consequently impacting the model’s accuracy. This further confirms that augmenting the model’s parameters and depth does not inherently result in improved performance in low-complexity visual classification tasks, such as cattle back pattern identification.

**Figure 10:**
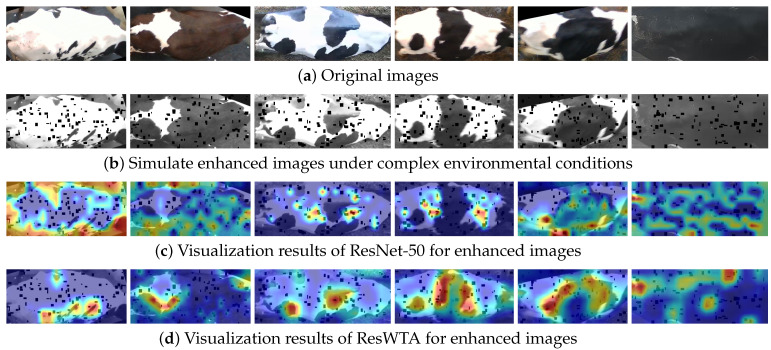
Visual heatmap analysis of model’s feature extraction capabilities in complex environments.

To evaluate the model’s feature extraction capabilities in complex environments, this study first applies four types of enhancements to the original images: grayscaling, blurring, brightness and contrast adjustment, and random occlusion of small targets. The enhancements replicate production environment interferences, including nighttime infrared illumination, lens defocus, natural light variations, and contamination on the cattle’s body surfaces and lenses. Subsequently, LayerCAM generates the output heat maps for the ResNet50 and ResWTA models based on the enhanced data in Stage 4, as illustrated in Figure 10. A comparison with Figure 10c,d indicates that ResWTA exhibits superior focus on the overall pattern structure of the cattle’s back under the aforementioned interference conditions, demonstrating significantly better anti-interference capabilities than ResNet50. This verifies that the combination of low-frequency wavelet convolution and the spatial attention mechanism effectively enhances the model’s ability to extract overall pattern features and improve local anti-interference performance.

**Figure 11:**
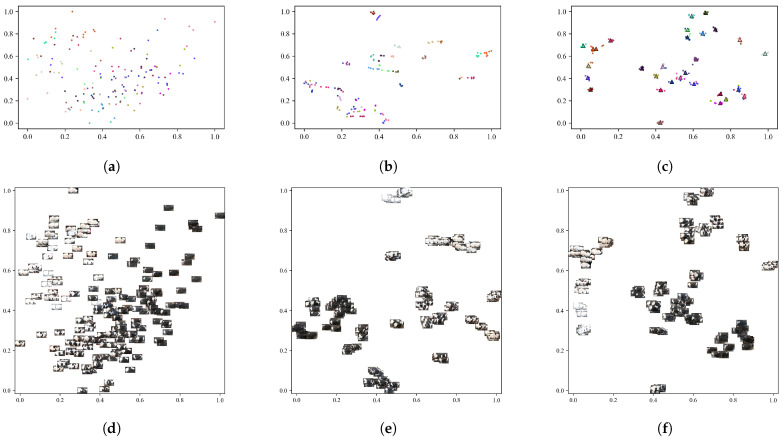
Analysis of t-SNE feature dimensionality reduction for the test set. Different color dots represent different individuals. (**a**) Results of ResWTA for t-SNE image visualization of the test set before training; (**b**) results of ResWTA combined with cross-entropy loss for t-SNE image visualization of the test set after training; (**c**) results of ResWTA combined with Triplet loss for t-SNE image visualization of the test set after training; (**d**) results of ResWTA for t-SNE visualization of the test set before training; (**e**) results of ResWTA combined with cross-entropy loss for t-SNE image visualization of the test set after training; (**f**) results of ResWTA combined with Triplet loss for t-SNE image visualization of the test set after training.

This study utilizes t-SNE dimensionality reduction visualization to assess the impact of Triplet loss on the generation of final discriminative features, employing one set of prototype samples (five samples) for each cattle in the test set. Figure 11 presents the visualization results. Analysis of Figure 11a,d reveals that the features extracted by ResWTA prior to training are scattered randomly in the low-dimensional space, with unclear category boundaries. Following the adoption of cross-entropy loss in ResWTA, the clustering of feature space becomes progressively clearer, as illustrated in Figure 11b,e, which aligns closely with the model’s visualization effects on the validation set. The introduction of Triplet loss in place of cross-entropy loss further enhances the discriminative nature of the features generated by the model, as depicted in Figure 11c,f. The features are more tightly clustered within classes and distinctly separated between classes. This improvement can be attributed to the ability of Triplet loss to bring similar samples closer together while pushing dissimilar samples apart, thereby enhancing classification performance, which aligns with existing research conclusions [35,44]. Additionally, this study employs the same-color triangles to label the prototype positions of the five samples, as shown in Figure 11c, indicating that the prototypes are highly representative of the samples and exhibit strong discriminability.

The results of the ablation experiments presented in Table 5 indicate that employing ResWTA for feature extraction enhances the model’s TOP-1 accuracy by 2.33 percentage points compared to ResNet50. Additionally, the introduction of the median prototype mechanism further increases the accuracy by 3.63 percentage points, while the implementation of the image enhancement strategy contributes an additional improvement of 1.14 percentage points to the TOP-1 accuracy. These findings substantiate that enhancements in the feature extraction network, the prototype mechanism, and the image enhancement strategy collectively improve incremental identification performance. Specifically, WTAConv significantly enhances the model’s capability to characterize the overall mottled contour of the cattle’s back by amplifying the representation of low-frequency and global features. The median mechanism demonstrates greater robustness to outlier samples during prototype construction, effectively mitigating noise interference and thereby enhancing category discrimination stability. Moreover, the image enhancement strategy bolsters the model’s adaptability to environmental interferences, such as light fluctuations and occlusions, by expanding the training sample space. The synergistic effect of these three strategies systematically improves the model’s generalization ability and robustness in open incremental identification scenarios.

Table 7 summarizes the principal types of identification errors observed in the test set during the experiments, including high pattern similarity, inconspicuous features, and extensive occlusion. Table 7(a) shows errors caused by high pattern similarity. The low-frequency wavelet convolution (WTAConv) adopted in this study effectively enhances low-frequency features and improves the overall representation of pattern structure. However, as the model does not explicitly model high-frequency details, some critical high-frequency cues may not be fully utilized, resulting in certain limitations when distinguishing individuals with highly similar patterns. This phenomenon is consistent with the perspective of Fan et al. (2023), who emphasized that only by fully leveraging the complementarity of high- and low-frequency information can the discrimination accuracy of object edges and details be significantly improved [45]. Table 7(b),(d),(e) show errors caused by inconspicuous features (i.e., sparse patterns or nearly solid coloration), where the root cause is the limited discriminative features extractable by the model from such individuals. Previous studies have demonstrated that the distinctive black-and-white patterns of Holstein cattle are the primary visual basis for individual identification, and mainstream methods in feature extraction and modeling are highly dependent on this information [1,15]. Therefore, when the pattern features of the target individual are insufficiently prominent, the identification performance is significantly affected. Table 7(c) presents errors caused by extensive occlusion, where the absence of key feature regions prevents the model from acquiring sufficient discriminative information, leading to identification failure.

In conclusion, while the proposed model in this study effectively enhances the extraction and differentiation of Holstein cattle back features, there remains a risk of misidentification for individuals that exhibit high similarity, sparse features, or significant occlusion. The model enhances identification accuracy through the incorporation of multi-level wavelet transforms and spatial attention modules; however, this improvement is accompanied by an increase in training and inference time costs, resulting in a relatively high deployment cost on edge devices in agricultural settings. Future research will concentrate on the integration of RGB-D multimodal image data of Holstein cattle, examining identification techniques in scenarios characterized by high similarity, sparse features, and extensive occlusion, while also pursuing lightweight model development to improve applicability in actual farm settings.

## 6. Conclusions

This paper addresses the challenges associated with the dynamic integration of Holstein cattle in production environments and proposes a ResWTA feature extraction network alongside a prototype network. These components are combined with the Triplet loss function and Manhattan distance metric to develop a high-accuracy incremental identification framework for individual Holstein cattle. The framework attained an accuracy rate of 94.33% in the identification of 600 sets of positive and negative sample groups derived from 30 Holstein cattle across three distinct production environments. In comparison to the baseline model, the model exhibited a 4.89% reduction in forgetting rate and a 2.4% enhancement in average incremental accuracy. This study presents improved identification robustness and incremental identification performance compared to current open-set identification models, offering an effective solution for the intelligent management of Holstein cattle. Simultaneously, evaluating the model’s scalability across various application scenarios will significantly enhance the adoption of machine vision technology in incremental identification contexts.

## Figures and Tables

**Table 1:** Sample distribution of the dataset.

Dataset	Number of Cattle	Number of Images
Train Dataset	272	33,775
Val Dataset	272	8443
Test Dataset	30	3940
Total	302	46,158

**Table 2:** Experimental parameter setting.

Hyperparameters	Value
Input resolution	224 × 224
Batch size	64
Training epochs	50
Optimizer	SGD
Base learning rate	0.1
Momentum	0.9
Weight decay	1 × $10^{-4}$
LR schedule	MultiStep (30th epoch)
Evaluation metrics	Acc@1/5, Precision/Recall/F1-score

**Table 3:** Comparison of classification performance of different feature extraction networks.

Model	Top-1 Accuracy (%)	Top-5 Accuracy (%)	Params (M)	Training Time (s)
MobileNet-V2	91.00	97.29	3.50	1513
ShuffleNet-V2	92.18	97.32	**2.28**	2022
EfficientNet-V2	92.05	97.83	7.14	2045
ResNet18	91.13	96.80	11.69	**1068**
ResNet34	93.24	97.65	21.18	1598
ResNet50	94.41	97.87	25.56	1807
ResNet101	93.68	97.71	44.55	2624
ResNeXt101	91.89	98.02	88.79	3075
ResWTA	**97.43**	**99.54**	30.70	2059
ResWTA-101	96.40	99.38	49.69	2391
ResWTA-X101	94.91	98.69	93.93	3165

**Table 4:** The experimental results for incremental identification.

Model	Loss Function	Prototype Method	Distance Metric	Top-1 Accuracy (%)
ResNet50	Triplet	Median	Manhattan	92.47
ResWTA	Triplet	Median	Manhattan	94.33

**Table 5:** Results of the ablation experiment on core variables.

Model	Loss Function	Image Enhancement	Prototype Method	Distance Metric	Top-1 Accuracy (%)
ResNet50	Triplet	-	-	Manhattan	87.23
ResWTA	Triplet	-	-	Manhattan	89.56
ResWTA	Triplet	No	Median	Manhattan	93.19
ResWTA	Triplet	Yes	Median	Manhattan	94.33

**Table 6:** The F and AIA for incremental identification.

Model	F (%)	AIA (%)
Framework with ResNet50	7.13	92.7
Framework with ResWTA	**2.24**	**95.1**

**Table 7:** Examples of identifying errors.

Label	Image	gt_label	pred_label	pred_image	Cause
(a)	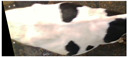	cattle_25	cattle_151	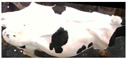	High pattern similarity
(b)	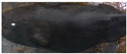	cattle_5	cattle_168	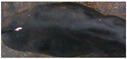	Inconspicuous features
(c)	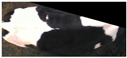	cattle_52	cattle_57	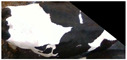	Extensive shelter
(d)	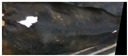	cattle_30	cattle_178	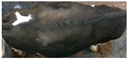	Inconspicuous features
(e)	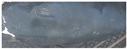	cattle_119	cattle_257	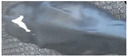	Inconspicuous features

## Data Availability

The dataset related to this study is published on GitHub repository https://github.com/bobdevops/Holstein-Incremental-ID, accessed on 5 August 2025.

## Abbreviations

The following abbreviations are used in this manuscript:

SSIM	Structure Similarity Index Measure
WT	Wavelet Transform
IWT	Inverse Wavelet Transform
LL	Low-Low Frequency Component
LH	Low-High Frequency Component
HL	High-Low Frequency Component
HH	High-High Frequency Component
SA	Spatial Attention
CBAM	Convolutional Block Attention Module
SGD	Stochastic Gradient Descent
TP	True Positive
FN	False Negative
IIF	Incremental Identification Framework
AIA	Average Incremental Accuracy
F	Forgetfulness
